# Identifying research priorities with children, youth, and families: A scoping review

**DOI:** 10.1177/13674935231151748

**Published:** 2023-01-16

**Authors:** Shokoufeh Modanloo, Quinn Correll, Rhonda Correll, Nathalie Major, Michelle Quinlan, Jessica Reszel, Jodi Wilding, Zhi Lin Zhou, Linda S Franck, Denise Harrison

**Affiliations:** 1 Arthur Labatt School of Nursing, Faculty of Health Sciences, 70383 Western University, London, ON, Canada; 2 Rankin School of Nursing, St Francis Xavier University, Antigonish, NS, Canada; 3 Children’s Hospital of Eastern Ontario 274065 (CHEO) Research Institute, Ottawa, ON, Canada; 4 School of Nursing, 8783 University of California, San Francisco, CA, USA; 5 Department of Nursing, School of Health Sciences, Faculty of Medicine, Dentistry and Health Sciences, University of Melbourne, Melbourne, VIC, Australia

**Keywords:** (MeSH terms) Research, child, adolescent, patient participation, health priorities, patient-centered care

## Abstract

Increased patient advocacy has resulted in a shift toward more active patient engagement in the research. A scoping review was conducted to explore the literature on healthcare research priority settings wherein children, youths, or their families were involved in the priority-setting process. Six databases including MEDLINE, CINAHL, PsycINFO, Embase, Web of Science, and Global Health and the James Lind Alliance website were searched up until September 2019. All primary studies involving children (<18 years of age) or families in developing research priorities in health care were included. All retrieved references were uploaded into Covidence, and two independent reviewers screened the search results. Descriptive thematic analysis was used to identify common themes. A total of 30 studies with 4247 participants were included. Less than half of the participants (*n* = 1237, (33%) were pediatric patients and their families. A total of 455 research priorities were identified. Three common themes emerged: (i) quality of care delivery, (ii) self-efficacy in health behaviors, and (iii) community engagement in care. This scoping review revealed priority research health topics from the perspectives of children, youths, or their families. The findings may be used as a foundation for future research to improve the health outcomes of children, youths, or their families according to their identified priorities.

## Introduction

There has been increased patient advocacy research focused on the interests and needs of the community over the past few years, resulting in a shift toward more active patient involvement initiatives in research (Boivin et al., 2018; Manafò et al., 2018). Some examples of these initiatives are patient engagement in setting research priorities (the James Lind Alliance (JLA)) (The James Lind Alliance, 2020), patient involvement in the development of patient-reported outcome measures (PROM) (Wiering et al., 2017), Patient-Centered Outcomes Research Institute (PCORI) (PCORI, 2020), INVOLVE Advisory Group (INVOLVE, 2020), and patient-oriented research by Canadian Institutes of Health Research (CIHR). Strategy for Patient-Oriented Research (SPOR) SUPPORT (Support for People and Patient-Oriented Research and Trials) units aim to promote patient and public engagement in the research process (SPOR SUPPORT Units - CIHR, 2020). Yet, patients’ and families’ values, experiences, and perspectives may not be sufficiently incorporated into health research (INVOLVE; PCORI; Rolfe et al., 2018).

While researchers have expertise in their biomedical focus area as well as academic expertise in research design and methods, patients and their families bring another level of expertise through their lived experiences with a condition or illness (SPOR SUPPORT Units - CIHR, 2020). Research priority-setting exercises can be seen as a form of participatory research, where new ideas and research questions are determined through collaborations between patients and families with researchers, healthcare professionals (HCPs), healthcare organizations, and research funders (SPOR SUPPORT Units - CIHR, 2020).

Two previously published systematic reviews of research priority setting focused on the care of children with chronic diseases (Odgers et al., 2018) and life-limiting conditions (Booth et al., 2018) included 83 and 24 priority-setting reports, respectively. They highlighted the importance of engagement of children, youths, and their families. Yet, most current research approaches entail a few inputs from patients and their families which may result in research output that does not answer questions that matter most, or even at all, to the patient population (INVOLVE; PCORI; Rolfe et al., 2018). This lack of relevance can lead to poor uptake of knowledge or effective translation of findings into clinical practice (Marlett et al., 2015). Given the vulnerability of sick children and their families, and the need for sensitive processes to obtain consent for research participation, the involvement of children and families in setting the research agenda is necessary and timely and may save resources otherwise spent on research considered unimportant to families (Contopoulos-Ioannidis et al., 2010).

**Aim.** To systematically identify and review existing research priorities in pediatric research wherein children, youths, or their families were involved in the priority-setting process.

## Methods

### Research design

This scoping review followed Arksey and O’Malley framework (Arksey and O’Malley, 2005) which is widely used in health research studies for scoping reviews (Munn et al., 2018). Scoping review is a preferred approach to identify the nature and extent of interdisciplinary evidence that may inform policy and decision-making in practice (Grant and Booth, 2009). This design helps researchers to map a diverse body of literature, provide an overview of a topic, clarify key concepts, and examine the characteristics or factors related to the concept (Munn et al., 2018). The suggested five stages of a scoping review (Arksey and O’Malley, 2005) used in this study are outlined as follows.

#### Stage 1. Identifying the research question

The purpose of this scoping review was to identify and summarize the existing literature on research priorities wherein children, youths, or their families were involved in research process across different health conditions.

#### Stage 2. Identifying relevant studies

For a study to be included in this scoping review, it was required to be a primary study, published in English, with a focus on health, illness, and/or healthcare, related to setting research priorities in any pediatric healthcare setting, no matter the country or origin. It also needed to include children or youths (<18 years of age) or parents/guardians of neonates, infants, children, or adolescents under the age of 18 years in setting the research priorities in healthcare settings. No restrictions were applied in terms of inclusion criteria for different methods of study. Non-primary studies such as literature reviews and synthesized studies were excluded.

#### Stage 3. Study selection

A comprehensive search strategy was completed in six scientific databases, as well as grey literature and reference lists of included studies. In the first step, a systematic search strategy was developed for each database in partnership with a health sciences librarian. MEDLINE (Medical Literature Analysis and Retrieval System Online), CINAHL (the Cumulative Index to Nursing and Allied Health Literature), PsycINFO (Psychological Information Database), Embase (Excerpta Medica database), Web of Science, and Global Health were searched with the developed search strategies. Our search strategy is listed in a Supplementary file (Appendix A). In addition, for the purpose of grey literature, the James Lind Alliance website (The James Lind Alliance, 2020) was searched for any additional resources. Finally, reference lists of included studies, as well as any relevant systematic reviews or literature reviews, were screened further for additional studies.

#### Stage 4. Data extraction

All references were uploaded into Covidence (Covidence systematic review software, Veritas Health Innovation, Melbourne, Australia), a web-based software produced to assist in the screening process of systematic and scoping reviews (https://www.covidence.org/home). In initial phase, titles and abstracts of all articles were screened for eligibility according to the inclusion criteria. Next, full texts of articles were retrieved and reviewed for eligibility. Finally, a list of included studies was generated. All parts of screening were performed by two reviewers independently (SM and QC), with any discrepancies discussed until a consensus was reached. In the chance that a consensus could not be determined, a third party was consulted (DH).

#### Stage 5. Collating, summarizing, and reporting the results

A single reviewer (QC) independently extracted data from all included studies and entered data into an Excel spreadsheet. A second reviewer (SM) verified the extracted data for accuracy and missing information. The information in a spreadsheet was organized into headings, including the year of publication, authors, country where the study was conducted, population of study, sample size, study design, health topic, research priorities identified, and the contribution of family and/or child, youth’s engagement.

To identify common themes in families’, children’s, and youths’ contributions and research priorities, a descriptive thematic analysis was used (Braun and Clarke, 2006). Thematic analysis was an iterative process by authors and involved independent coding, data display, recoding, and verification. The themes were reviewed multiple times, and revisions were made to the labelling and organization of the themes and sub-themes. We started by reading the research priorities and statements describing the contribution of family and/or child’s engagement and coded data into the higher-level coding matrix. Inductive themes were created under each category or attribute.

## Results

### Study characteristics

**Figure 1.**
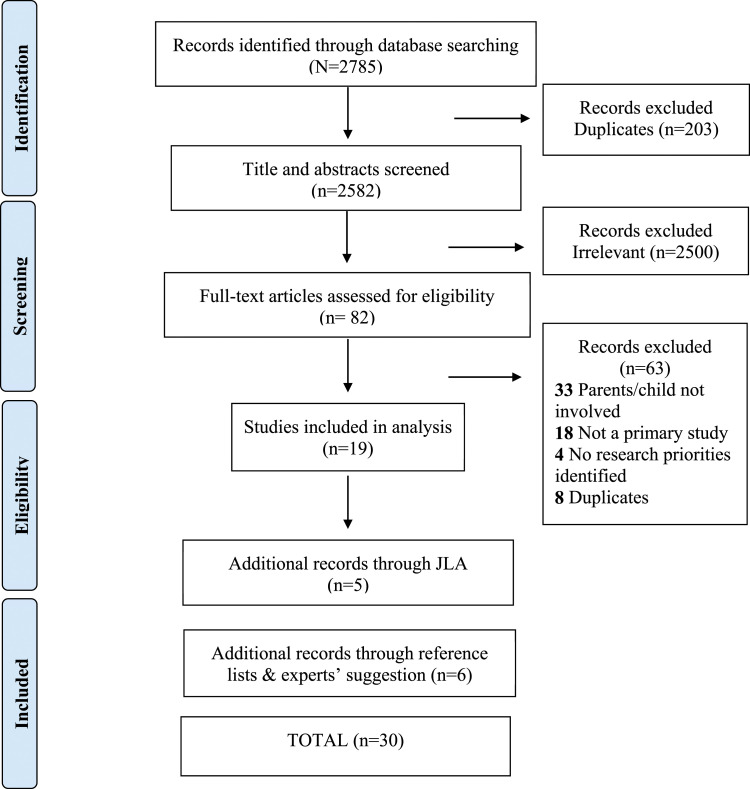
PRISMA flow chart.

Databases were searched systematically on 19 September 2019. As shown in Figure 1, initial search results identified 2758 records, and after removing duplicates, 2582 (93%) titles or abstracts were screened for eligibility. Of these, 2500 (97%) were considered irrelevant to the topic, leaving 82 (3%) full-text studies, which were assessed for eligibility. A total of 19 studies (23%) were included in the analysis after removing further studies due to duplication (*n* = 8, 10%); no children, youths, or family members were involved (*n* = 33, 40%), not primary studies (*n* = 18, 22%), no research priorities identified (*n* = 4, 5%). A further five studies were added by a manual search of the JLA database, and a further six studies were added following searching reference lists and experts’ suggestions, bringing the total included records to 30 studies in the final analysis *(PRISMA flowchart is presented in* Figure 1*).*

The majority of studies took place in a single country (*n* = 28, 93%). Most studies were conducted in the United Kingdom (UK) (*n* = 10, 34%), the USA (*n* = 9, 30%), and Australia (*n* = 5, 17%). Most studies were published in the last 5 years (*n* = 21, 70%), and the remaining studies were published in the last two decades (earliest 1995, latest 2019). Four (13%) studies were focused on pain and palliative care, and 4 (13%) were focused on the fields of hematology, oncology, immunology, or infectious diseases. The remaining studies covered a wide range of health conditions affecting pediatric populations. Characteristics of included studies are presented in Table 1.

**Table 1.** Characteristics of included studies.

Health topic	Country (Year)	Sample size (N = total) (n = subset of the sample)	Study method	Family/child contribution (as reported)
Traumatic injuries (Lash et al., 1995)	USA (1995)	Families (*n* = 67)	Questionnaire	Highlighted the important issues for families
Hematology, oncology, immunology, and infectious diseases (Soanes et al., 2000)	USA (2000)	• HCPs (*n* = 16) • Parents (*n* = 10)	Questionnaire	• Highlighted patient/family-centered priorities • Involved valuable experience of families
Hospice care (Malcolm et al., 2008)	UK (2008)	• Staff/volunteers (*n* = 44) • HCPs (*n* = 18) • Families (*n* = 5)	Interview and focus group	• Included the personal situation about the child’s illness and the resultant impact on their family • Highlighted the importance of tailoring the services to meet their specific care and support needs • Understood the views and experiences of children and families
Hospice care (Malcolm et al., 2009)	UK (2009)	• Families (*n* = 293) • Staff/volunteers (*n* = 216) • HCPs (*n* = 112)	Delphi survey (3-round)	Provided insight into the perspectives of service users and highlighted their roles as active participants in the following • Decisions affecting their care • Development of an extensive research agenda • Incorporating the issues that matter most to service users
Cerebral palsy (McIntyre et al., 2010)	Australia (2010)	• Consumers (not specified) (*n* = 20) • Researchers (*n* = 31) • HCPs (*n* = 76)	Delphi survey (3-round)	Not reported (NR)
Hematological cancer (Clinton-McHarg et al., 2010)	Australia (2010)	• HCPs • Researchers and teachers(*n* = 112) • Patients and carers (*n* = 45)	• Expert advisory group • Web-based survey	Provided additional perspectives about the impact of the illness and its treatment
Cleft lip and palate (the James Lind Alliance, accessed 2020)	UK (2012)	Patients, carers/parents andHCPs (N = NR)	• Questionnaire • Workshop	NR
Developmental-behavioral (Blum et al., 2012)	USA (2012)	• Pediatricians (*n* = 27) • Psychologists (*n* = 16) • Parents (*n* = 12)	Delphi survey (3-round)	Prioritized research related to the daily challenges of families in interacting with service systems, such as family support, family-centered care, and decision-making
Epilepsy (Berg et al., 2013)	USA (2013)	Parents, representatives of voluntary advocacy organizations, HCPs, researchers, and administrators (N = NR)	• Group discussion • Workshop	• Created a better platform for parents to communicate • Engaged parents more explicitly as a part of the decision-making and treatment team
Palliative care (Baker et al., 2015)	USA (2015)	• Experts (*n* = 170) • Parents (*n* = 72)	Delphi survey (4-round)	• Indicated how best to prepare patients and their families • Warranted pediatric-specific quality indicators
Neurodisability (Morris et al., 2015)	UK (2015)	Survey • Parents (*n* = 26) • Young person (*n* = 1) • Charity representatives (*n* = 10) • HCPs (*n* = 39) Workshop • Young people (*n* = 3) • Parents (*n* = 7) • Charity representatives (*n* = 3) • HCPs (*n* = 9)	• Focus group • Survey • Workshop	• Included the views of children and young people • Highlighted the importance of tailoring and evaluating public health guidance to be more appropriate for children and young people
Cancer (Medlow and Patterson, 2015)	Australia (2015)	• Consumers (patients, survivors, parents, and siblings) (*n* = 26) • HCPs (*n* = 75)	• Literature reviewing • Experts consulting • Online survey	NR
Attention deficit hyperactivity disorder (ADHD) (Jacobson et al., 2016)	Sweden (2016)	• Parents (*n* = 7) • HCPs (*n* = 7)	Working group	NR
Pediatric obesity (McPherson et al., 2016)	USA (2016)	• Researchers (*n* = 12) • Trainees (*n* = 4) • HCPs (*n* = 12) • Parents (*n* = 3) • Former clients with disabilities (*n* = 1) • Community partners (*n* = 3) • Decision makers (*n* = 3)	• Group discussion • Workshops	• Ensured timely and meaningful research for knowledge users • Integrated children’s and families’ needs • Highlighted the importance of guideline development on how best to engage children/families meaningfully in designing both clinical interventions and health promotion research initiatives
Rheumatic disease (Parsons et al., 2016)	UK (2016)	Patients (*n* = 63)	Focus groups	• Provided a variety of flexible ways for people to share their views and experiences • Ensured the patients’ voices as the heart of our work
Preventative care (Lavigne et al., 2017)	Canada (2017)	Survey • Parents (*n* = 115) • HCPs (*n* = 42) Workshop • Parents (*n* = 10) • HCPs (*n* = 18)	• Survey • Literature review • Workshop	Balanced the viewpoint of stakeholders
Pain and palliative care (Liossi et al., 2017)	UK (2017)	• HCPs (*n* = 11) • Parent representative (*n* = 1)	• Brainstorming session • Teleconference discussion	Allowed individuals the opportunity to voice their opinions
Sudden infant death syndrome (SIDS) (Hauck et al., 2017)	25 countries (2017)	Surveys: (*N* = 691) (NR) Workshops • HCPs (*n* = 8) • Researcher (*n* = 14) • HCPs • Family (*n* = 12) • Medical examiner (*n* = 2) • NGO representative (*n* = 5) • Others (*n* = 3)	• Surveys (2-round) • Workshops	NR
Systemic lupus erythematosus (SLE) (Tunnicliffe et al., 2017)	Australia (2017)	Patients (*n* = 26)	• Focus group • Interviews	Highlighted the importance of • Creating culturally relevant resources for patients and families • Minimizing family/community burden • Informing a patient-focused research agenda • Improving treatment satisfaction, health, and quality of life outcomes
Emergency medicine (Bialy et al., 2018)	Canada (2018)	• Parents (*n* = 11) • Emergency physicians, nurses, administrators, educators, and trainees (*n* = 96)	• Survey (3-round) • Meeting	• Enabled a rich dialogue about the context and importance of the topics from a healthcare user’s point of view • Making the patient and caregiver perspectives explicit can change the focus of research and result in changes to outcomes, goals, and improvement of measurement tools • Helped in identifying patient-centered outcomes and co-design studies in these priority research areas
Post-pediatric ICU (Manning et al., 2018)	UK (2018)	• Patients (*n* = 8) • Parents (*n* = 6) • HCPs (*n* = 8) • Commissioner (*n* = 1) • Service manager (*n* = 1)	Discussion group	• Highlighted the direction for future services, interventions, and research to be holistic and family-centered • Highlighted the importance of family support and the unmet needs of siblings
Cancer (Aldiss et al., 2018)	UK (2018)	• Patient representatives (*n* = 5) • Coordinating team (*n* = 4) • HCP representatives (*n* = 9)	• Survey • Group discussion • Workshop	NR
Cystic fibrosis (Rowbotham et al., 2018)	23 countries (2018)	*N* = 482 respondents [NR)	• Surveys (2-round) • Systematic review • Workshop	• Invigorated research in areas of shared importance to both the patients and the clinical community • Demonstrated the value of involving the community in all steps of the research pathway
Lower limb surgery (the James Lind Alliance, accessed 2020)	UK (2018)	Survey • Children, parents/carers and HCPs (*n* = 234) Workshop • Individuals (*n* = 30) • Physiotherapists (*n* = 6) • Parents (*n* = 9) • Patients (*n* = 4) • Orthopedic surgeons (*n* = 7) • Clinical scientist (*n* = 1) • Advanced nurse practitioner (*n* = 1) • Charity representatives (*n* = 2)	• Survey • Workshop	NR
Preterm birth (Franck et al., 2018)	USA (2018)	Individuals from the homeless prenatal program (*n* = 6) and Black Infant Health Program (*n* = 9)	Facilitated group work (2 focus groups)	NR
Children and Youth with Special Health Care Needs (CYSHCN) (Coller et al., 2020)	USA (2018)	Survey • Children or family members (*n* = 64) • Health professionals, administrators and, policy experts, AND researchers (*n* = 2005) Expert panel: (*n* = 7)	• Delphi survey (2-rounds) • Literature • Expert panel	NR
Inflammatory bowel disease (Grant et al., 2019)	Canada (2019)	Survey • Patients (*n* = 25) • Caregivers (*n* = 20) • HCPs (*n* = 30) Workshop • Patients (*n* = 2) • Caregivers (*n* = 4) • Sibling (*n* = 1) • HCPs (*n* = 5)	• Workshop • Surveys (2 rounds) • focus groups • Review of clinical practice guidelines	• Represented pediatric patient voice • Helped in improving the quality of research • The materials produced are of value to patients and in a format that will have good uptake for those interested • Helped in identifying symptoms that have the most impact on patients, identifying true treatment uncertainties, to improving the likelihood of recruiting to target
Patient safety (Hoffman et al., 2019)	USA (2019)	Survey • Nurse leader (*n* = 14) • Physician leader (*n* = 16) • Pharmacist (*n* = 2) • Researcher (*n* = 5) • Parent (*n* = 48) • Non-clinical QI leader (*n* = 19) Interview: Health system executives (*n* = 7)	• Survey (2 rounds) • Interviews	NR
Chronic conditions (Lopez-Vargas et al., 2019)	Australia (2019)	• Patients (*n* = 3) • Parents (*n* = 19) • Representatives from consumer organizations (*n* = 13) • HCPs (*n* = 38)	Group discussion workshop	• Helped in the alignment of research investment with the priorities of children with a chronic condition and their caregivers • Understood children and their families gave higher priority to psychosocial health and non-pharmacological interventions than health professionals • Highlighted the importance of research on improving health literacy so patients and parents could be well-informed, empowered, and able to be partners in their care • Strengthened the ability to cope and impact families
Learning difficulties (Lim et al., 2019)	UK (2019)	Surveys • Individuals (*n* = 41) • Parents/carers (*n* = 125) • HCPs (*n* = 195) Workshops • Young people (*n* = 5) • Parents (*n* = 6) • HCPs (*n* = 14)	• Survey (2 rounds) • Systematic reviews • Workshops	Identified unanswered questions that shape research and influence practice and policy

^a^NR = Not reported.

### Participants

A total of 4247 participants engaged in identifying research priorities across 28 included studies, and the number of participants was not reported in two studies. In 25 of the 28 (89%) studies (which included 3765 participants), the number of participants from different stakeholders (i.e., families, children, youths, HCPs, and researchers) was distinguished, in which two-third of the participants (*n* = 1237, 33%) were pediatric patients and their families. The remaining participants (*n* = 2528, 67%) were identified as HCPs, researchers, volunteers, representatives of organizations, decision-makers, coordinators, administrators, and community partners (Table 1). Most studies included more than one type of participant (*n* = 26, 87%), with a combination of families and HCPs being the most common pairing.

### Research priority setting methods

Eighteen (60%) of the studies used more than one method to conduct their research priority setting. The combination of using a survey followed by a group discussion or one-to-one interviews was used in 14 (47%) studies. Group discussion and one-to-one interviews were used together in three studies (10%). Other methods included one-time surveys (*n* = 2, 7%) or rounds of Delphi survey (*n* = 4, 13%) and only group discussion (i.e., workshops and focus groups) (*n* = 7, 23%). Delphi method surveys were used in 11 studies (37%) to reach consensus including 2 (*n* = 6, 54%), 3 (*n* = 4, 36%), and 4 rounds (*n* = 1, 9%) of surveys. In addition, 3 (10%) of the studies performed a literature review prior to commencing the research priority consensus procedure, with the purpose of eliciting the initial questions/priorities in research area. Further details of each study method are listed in Table 1.

### Synthesis of research priorities

A total of 455 research priorities were identified from the 30 included studies. Studies commonly identified a list of top 10 research priorities. The research priorities identified were grouped into three common themes: i) quality of care delivery, ii) self-efficacy in health behaviors, and iii) community engagement in care. Quality of care delivery included sub-themes of prevention, diagnosis, treatment, and symptom management; patient safety and infection control; access to and navigation of health care service. Self-efficacy in health behaviors included the sub-themes of mental health care, healthy lifestyle and quality of life, and communication (family–child–HCPs). Community engagement in care included the sub-themes of school support, access to local community resources, family support, and transition-in-care support. A list of the top 20 research priorities identified across the studies is presented in Table 2. The top priorities are extracted based on their commonality and repetition in the studies. No specific outstanding theme was recognized by considering different age groups, countries, or health conditions. A complete list of identified research priorities and the list of codes, themes, and sub-themes are presented in Supplementary file Appendixes B and C.

**Table 2.** Top 20 research priorities.

Improving pain management and sedation practice
Evaluating different parents’/siblings’/partners’ support programs following the child’s diagnosis, treatment, or death to improve family functioning and coping strategies
Evaluating the role of diets, weight control, and engagement in physical activity in the management of childhood disease and obesity prevention
Integration of community organizations with childcare delivery and improving access to community resources for families and children outside the medical system
Improving learning and educational outcome of children with health problems at school with teacher support
Evaluation of effective strategies for screening and prevention of mental health problems and better access to mental health support to improve children’s psychological well-being and social functioning
Raising awareness of childhood health conditions in communities, schools, and workplaces
Using child-centered strategies to improve patients’ self-efficacy and quality of life and reduce lifestyle disruption after a health condition
Manage, maintain, and optimize patient/family adherence with recommendations or treatment
Support during transition from hospital to home care
Support during transition from pediatric to adult care
Understanding social, cultural, and environmental risk factors for a childhood health condition
Effectiveness of different therapy interventions with fewer short- and long-term side effects for children
Implementing family-centered care initiatives with the early and sustained engagement of families
Develop and evaluate strategies to help families make difficult care decisions
Use of online interventions/technology information/electronic applications that help patients/families in promoting self-management
Screening and early and accurate diagnosis of childhood diseases
Improving patient safety and infection control practices in hospital settings
Optimize communication and information sharing between parents/carers and professionals
Identify biological (biomarkers) and genetic factors and causes of childhood diseases

### Children, youths, and family contributions

Studies were reviewed further for more information regarding reflections or statements made by authors on what the inclusion of children, youths, and/or families resulted in regarding outcomes of the research priority-setting exercises. In general, these statements fell under three main categories: (i) incorporating needs, values, and experiences of families, (ii) creating resources to meet family and/or child’s needs and tailoring the service accordingly, and (iii) empowering family and/or child to be a partner in care and care decision-making. Summaries of the statements are listed in Table 1.

## Discussion

The aim of this scoping review was to systematically identify and review existing research priorities in pediatric research wherein children, youths, or their families were involved in the priority-setting process. Even though the inclusion criteria in this scoping review was inclusion of children, youths, and/or their families in the research priority setting, most participants were found to be researchers or HCPs.

In a systematic review of setting research priorities in 83 studies with a focus on childhood conditions with chronic diseases, parents were only included in 20 (24%) studies and children in only 4 studies (5%) (Odgers et al., 2018). This inconsistent engagement of children and families in research priority-setting process may be due to the relatively new focus of including patients and their families, as well as the lack of knowledge and skills of researchers in knowing how to involve patients and families in research (SPOR SUPPORT Units - CIHR, 2020). Other barriers may include a lack of support and funding for families and children to be involved and challenges for children, youths, and families in being able to articulate their ideas in research development (McDonagh and Bateman, 2012).

In this scoping review, many research priorities were identified across various childhood conditions. However, there were many overlapping priorities showing the commonality of research topics that are of importance for children, youths, and families. Most of the identified topics were about improving access to health care services, patient safety, infection control, disease prevention, early diagnosis, and improving treatment services in clinical health care settings for children. These results are consistent with the topics prioritized in adult studies (Manafò et al., 2018), particularly regarding the need for the provision of best practices including quality care delivery in treatment, symptom management, and disease prevention.

Research priority topics identified in this study were not limited to only clinical-specific aspects of health. Additionally, they showed the paramount role of psychosocial determinants of health in predicting the quality of life for children, youths, and their families. Examples included improving healthy lifestyles, better mental health care services, and better engagement of school, family, and local community resources in child health care. Some unique priorities identified in our review were considerations about transition to adult care, transition from hospital to home, communication with HCPs, access to community resources, and improving normality of everyday life (i.e., self-management, diet, exercise, and school).

The systematic review of research priorities in childhood chronic diseases (Odgers et al., 2018), which included 83 studies, showed that most research priority topics identified were focused on clinical aspects of care delivery such as treatment (65, 78%), disease trajectory (40, 48%), disease onset/prevention (36, 43%), prevalence (25, 30%), and diagnostic methods (23, 28%). In addition, they reported that themes such as quality of life and psychological impact (40, 48%), knowledge/self-management (27, 33%), access to healthcare (20, 25%), and transition to adulthood (10, 12%) were important areas requiring further research in childhood chronic disease (Odgers et al., 2018). Two recently published research priority setting studies with children, youths, and their families, focusing on children and youths with special health care needs (CYSHCN) (Coller et al., 2020) and chronic illnesses (Scheven et al., 2020), found similar high priority research topics. These included health care systems and health care coverage; communication between patients, parents, and HCPs; family and social support; and transition to home and to adult care.

Another important priority that emerged in this study was exploring children’s, youths,’ and families’ needs and engaging them in the care process and decision-making. A systematic review of 12 studies was conducted by Bray et al. (2018) to synthesize and examine the current evidence about methods to engage children and youths in healthcare consultations. Although this review study was not focused on setting research priorities, the findings are of high relevance as most of the reported interventions and methods were categorized as having a “low level of engagement” of children and youths and were mainly focused on providing information and instruction to encourage them to “talk more” or voice their concerns. Bray et al. showed that children’s engagement was shaped and controlled by adults in the consultation, and no child-led initiatives were identified in their review, with only fewer interventions directed toward developing skills in children and youths to become key reporters of their condition (Bray, et al., 2018). Involving families and children from outset needs to be considered as a priority in developing best practice guidelines, clinical interventions, and health research initiatives so that healthcare services can be directly tailored to meet their needs (Malcolm et al., 2009). Although patient-oriented initiatives have been recently established, the impact of patient engagement strategies is still unclear, given the time-consuming process of translating the research into practice (almost 17 years) (Morris et al., 2011). Consequently, the engagement of patients has been limited mostly to participating in the data collection phases of studies with little involvement in research priority setting, designing the studies, data analysis, or knowledge dissemination (Boivin et al., 2018; Manafò et al., 2018).

In line with these results, a scoping review of 207 studies on children participating in research between 2000 and 2018 in Australia showed that almost all studies included children only as a participant in the process of research with less input in other phases of the research process such as study design, developing research questions, data analysis, and dissemination of reports and findings (Grace et al., 2019). None of the studies in Grace et al.‘s review was related to setting research priorities with children. Engaging families or/and children and youths in setting research priorities will help ensure that the needs, experiences, and values of families are incorporated in health care planning, health care services, and resources are tailored accordingly to meet family and/or children’s needs (Boivin et al., 2018; Manafò et al., 2018). An optimal outcome is children, youths, and families are empowered to be equal partners in care decision-making. In addition, conclusions drawn from a systematic review of 24 studies on setting research priorities in life-limiting conditions among children were that no further priority-setting research is needed in this area unless families and children are engaged, and the perspective of families and children will be investigated (Booth et al., 2018).

### Limitations

The findings from this scoping review should be considered in light of its limitations. First, we excluded studies that did not focus on infants,’ children’s, or adolescents’ health problems or did not include them or their families in the process of research priority setting. However, it is possible that the excluded studies may have information about the perspectives of these patients or their families that we were unable to recognize in the process of screening in this study. In addition, as scoping reviews remain a newly emerged and evolving methodology in health sciences, the results of this study must be interpreted with caution due to limitations in study rigor in guiding the practice. We also acknowledge that our search only included studies published in English. In addition, most of the included studies were conducted in North America, the UK, and Australia. No data on the race and ethnicity of participants were collected due to a lack of documentation and availability of data specifying the racial and ethnic groups in included studies. This may affect the generalizability of results and highlights a need for similar research priority-setting studies focused on the health needs and priorities of marginalized families, children, and youths who may have different accessible resources and delivery of health care in underserved communities.

### Implication for practice

The needs of children, youths, and families are dynamically changing over time, and investment in research in this population is necessary to ensure the ongoing relevance of research with the aim of improving health outcomes. The identified research priorities in this study clarified a need for further research with the consideration of social determinants of health (i.e., roles of the education system, lifestyle, emotional support, and family or community support). It is recommended that researchers, HCPs and children, youths, and family partner promote the integration of patients’ priorities generated by children, youths, and families into the care.

## Conclusion

The results of this scoping review identified important research health topic areas from the perspectives of children, youths, and their families across diverse health conditions. It was revealed that children and their families were less represented in studies than other stakeholders. However, optimally, children and their families should be equal partners in setting priorities in research, along with other stakeholders. A research agenda aligned with these priorities may better lead to improvement in satisfaction for children, youths, and their families and may result in improved patient-centered research and overall quality care in future. The findings of this scoping review may be used as a foundation for future planning of research and may also guide funding organizations and policymakers to invest in prioritized research areas to improve the health outcomes of children, youths, and their families according to their identified research priorities.

## Supplemental Material

Supplemental Material—Identifying research priorities with children, youth, and families: A scoping review Supplemental Material for Identifying research priorities with children, youth, and families: A scoping review by Shokoufeh Modanloo, Quinn Correll, Rhonda Correll, Nathalie Major, Michelle Quinlan, Jessica Reszel, Jodi Wilding, Zhi Lin Zhou, Linda S Franck, and Denise Harrison in Journal of Child Health Care

## Abbreviations

JLA: The James Lind Alliance
UK: United Kingdom
PCORI: Patient-Centered Outcomes Research Institute
CIHR: Canadian Institutes of Health Research
KT: Knowledge Translation
SPOR: Strategy for Patient-Oriented Research
USA: United States of America
SUPPORT: Support for People and Patient-Oriented Research and Trials
HCPs: healthcare professionals
