# Supplementary material for: Identifying research priorities with children, youth, and families: A scoping review
Source: J Child Health Care. 2023 Jan 16;28(3):592–609. doi: 10.1177/13674935231151748 (PMC11459867; doi:10.1177/13674935231151748)
Supplement: Supplemental Material—Identifying research priorities with children, youth, and families: A scoping review [file sj-pdf-1-chc-10.1177_13674935231151748.pdf]

Appendix A. Search Strategy (MEDLINE)

MEDLINE	Date searched: 2019-05-27
1	exp Pediatrics/
2	adolescent/ or exp child/ or exp infant/
3	(infant disease* or childhood disease*).ti,ab,kf.
4	(babies or baby or child or girl or boy or infant or juvenile or kid or minors or neonate or neo-nate or newborn or new-born or paediatric or peadiatric or pediatric or perinate or preterm or preschool or pubescent or teen or toddler or youth).ti,ab,kw./
5	((pediatric* or paediatric* or infan* or child* or adolescen* or young).jn,jw. or (pediatric* or paediatric* or infan* or child* or adolescen* or young).in)
6	1 or 2 or 3 or 4 or 5
7	Research/
8	Patient Participation/
9	Health Priority/
10	7 and 9
11	8 and 9
12	(research adj3 (priorit* or lead* or preference* or prerogative* or agenda)). Ti,ab,kw.
13	10 or 11 or 12
14	family/ or parenting/ or parents/ or fathers/ or mothers/ or single parent/ or single-parent family/
15	Caregivers/
16	(parent* or caregiver* or mother* or father* or guardian* or family or families) ti,ab, kw.
17	exp Legal Guardians/
18	14 or 15 or 16 or 17
19	6 and 13 and 18
20	limit 19 to English Language

Appendix B. Identified Research Priorities Theme and Sub-themes, with Illustrative Codes

**Theme and Sub-Theme**		**Illustrative Codes**
Quality of care delivery	Prevention, diagnosis, treatment, and symptoms management	Pain management<br>Adherence to treatment<br>Long-term outcomes<br>Medication & Diagnosis errors<br>Screening & Early Detection<br>Comorbidity or complications<br>Biomarkers<br>Prognosis<br>Medical and surgical management techniques<br>Genetic basis of conditions<br>Efficacy of pharmacological treatments<br>Efficacy of behavioral interventions<br>Rehabilitation<br>Biologic or psychosocial related factors
	Patient safety and infection control	Quality improvement<br>Documentation<br>Shortfalls<br>Harm reduction<br>Evidence-based practice guidelines<br>Determinants of health<br>Informed consent<br>Environmental risk factors<br>Research methods innovations
	Access to and navigation of health care service	Decrease waiting time<br>Cost efficiency<br>Financing & insurance<br>Access to services and supports<br>Care planning & coordination service<br>Technology and care

		Follow-ups
Self-efficacy in health behaviours	Mental health care	Access to mental health service,<br>Emotional support<br>Improving coping strategies,<br>Psychosocial measures and support<br>Survivorship<br>Vulnerability, and complexity
	Healthy lifestyle & quality of life	Adherence with recommendations<br>Diet therapy and nutritional needs<br>Promote mobility & Engagement in physical activity<br>Live independently<br>Patients' needs and 'perspective
	Communication (family-child-HCPs)	Education<br>Supported conversations<br>Child-centered strategies<br>Help of technology and electronic applications<br>Communication improvement<br>Knowledge transfer
Community engagement in care	School support	Learning support<br>Education system impact<br>Integration of school and community<br>Teacher supports<br>Impact of stigma/ knowledge gaps/bullying<br>Promote social skills<br>Barriers to enter the workforce/going back to school
	Access to local community resources	Community and social support<br>Access to publicly available resources<br>Improve participation in community settings<br>Peer support/social activities
	Family Support	Engaging and partnership in care

		Exploring needs of siblings and families
		Empowerment for families
		Parental stress
		Parent-targeted educational tools
		Parenting methods
		Parental-child attachment
	Transition-In-Care support	Raising awareness
		Social and cultural factors
		Transition support from hospital to home
		Transition to adult care
		Decision making
		Caregiving at home

Appendix C. Identified Research Priorities (Supplementary data)

Health Topics	Research priorities
Traumatic childhood Injuries (Lash et al., 1995)	Emergency room treatment, emotional impact of treatment, parent-professional communication, learning and services of school/special educational programming, inpatient rehabilitation and therapies for muscles/movement and learning/memory, community resources, methods for families to get information
Cerebral palsy (CP) (McIntyre et al., 2010)	How can CP be prevented? What are the causes of and casual pathways to CP? What potential does the brain have to repair injury? Can stem cells have a therapeutic effect for CP? What policies are needed to improve quality of life for families? What factors have the greatest impact on improving the lifestyle and quality of life of individuals with CP? What are the barriers to employment that exist for people with CP? How can people with CP be better trained with the necessary skills to enter the workforce? Can people with CP equitably access the community? What is the relationship between CP and poverty?<br>What can be done to address the mismatch between what service parents and people with CP need and what they actually receive? What is the optimal intensity of therapy programs? What is the most efficient service model so that maximal services reach people with CP and their families? What are the optimal treatments for CP? What are the long-term outcomes of treatments? What is the effectiveness of alternative therapies for the treatment of CP? What early intervention (dependent on CP type) will prevent and minimize structural impairments? Does physiotherapy benefit people with CP? What are the most effective methods of pain management? What is the effectiveness of hydrotherapy for people with CP? What are the most effective methods of educating parents to help improve their child's independence and function? What is the impact of therapy type and duration on parents guided therapy by parents? Match aetiology ⁄ intervention prevention of a child with CP?
Developmental-Behavioral Pediatrics (DBP) (Blum et al., 2012)	Evaluate factors that facilitate the transition to adult health care, Identify child, family, or community factors that promote or interfere with family's ability to implement recommendations from DBP evaluations, Identify epigenetic changes brought about by stress and their effect on brain development, Identify biologic or psychosocial factors in preschool-age children that predict persistence of ADHD symptoms overtime, Determine the contribution of inadequate or irregular sleep patterns on children's psychological, Determine the stability of the ASD diagnosis over time for young children, Determine the nature and extent of social disability in children with developmental disorders other than ASD, Determine the prevalence of obesity in children with developmental disabilities and its relationship to pharmacological interventions, Test or technology assessment, Identify effective and efficient ways to measure functional impairment across developmental and behavioral diagnostic categories, Identify measures most sensitive to improvement in ASD

	symptoms over time, Evaluate methods of screening for ASD at young ages, Identify biological factors (biomarkers) useful in the diagnostic evaluation of children for ASD, Evaluate the impact of developmental-behavioral disorders on children's quality of life, Describe the mental health outcomes, risk, and protective factors for children with learning disabilities, Assess the impact of health behaviors such as physical activity and weight control on functional outcomes for children with developmental disabilities, Evaluate the efficacy of pharmacological treatments for children with ASD and comorbidities, Evaluate the efficacy or effectiveness of interventions for aggressive behavior in children , Identify protective factors and interventions to decrease mental health problems in children at high risk for mental health disorders, Evaluate whether cognitive behavioral therapy improves ASD symptoms in children with high functioning, Identify family, community, or biological factors that predict the effectiveness of behavioral or pharmacological interventions for children with ASD, Identifying pharmacogenetics variations that explain differences in efficacy or side effects of medications, Determine how to modify treatments in children who are not responding well to their ADHD treatment, Evaluate whether management of sleep disorders in children with ASD effects developmental or behavioral outcomes, Evaluate the efficacy of electronic applications for smart phones or other devices in promoting independence for individuals with disabilities, Determine how to improve social skills interventions for children with ASD so that they generalize to everyday situations, Evaluate the efficacy of behavioral interventions for ASD when delivered by parents or other nonprofessionals, Evaluate the efficacy of pharmacological treatments for children with ADHD and comorbidities, Define the long-term outcomes of behavioral treatments for ADHD, Evaluate the efficacy of cognitive behavioral therapy for the treatment of anxiety in youth with ASD, Evaluate whether the frequency of speech therapy effects language and communication outcomes for children with ASD, Compare the efficacy of treatment strategies for young children with ASD, Compare the efficacy of combined medication and behavioral or cognitive interventions with either one alone in children with developmental disorders, Compare pharmacologic and behavioral treatments for aggressive behavior in adolescents, Compare the effectiveness of different methods for teaching communication to nonverbal children with disabilities, Compare the effectiveness of interventions for insomnia in children with ASD, Evaluate strategies for teaching DBP to residents to prepare them for primary care practice, Evaluate children's understanding of their developmental behavioral diagnosis across ages and conditions , Evaluate the impact of a child's insurance coverage, Compare the efficacy of different family support programs to improve child and family functioning after a child is diagnosed with a developmental disability, Identify components of effective early intervention, Evaluate the feasibility and effect of implementing a family-centered care initiative, Evaluate the effect of strategies for including adolescents with disabilities in life decisions, on the decisions, and their outcome, Identify patterns of behavior change that indicate pain or ill health in nonverbal children with DDs, Describe the long-term side effects of atypical antipsychotics along with the ways to monitor for these effects , Evaluate the effectiveness of speech therapy for different developmental
Pediatric Epilepsy (Berg et al., 2013)	Patient outcomes, Early and accurate diagnosis and optimal treatment, Role and involvement of parents, Integration of school and community organizations with epilepsy care delivery, Resources outside the medical system

Children and young people with a neurodisability (Morris et al., 2015)	Does the timing and intensity of therapies alter the effectiveness of therapies? What is the appropriate age of onset/strategies/dosage/direction of therapy interventions? To improve communication for children and young people, Are child-centered strategies to improve children's (ie, peers) attitudes towards disability effective to improve inclusion and participation within educational, social and community settings? Does appropriate provision of wheelchairs to enable independent mobility for very young children improve their self-efficacy? Are counselling/psychological strategies effective to promote the mental health? What is the (long-term) comparative safety and effectiveness of medical and surgical spasticity management techniques? Does a structured training programme, medicines and/or surgery speed up the achievement of continence (either/or faecal or urinary)? What strategies are effective to improve engagement in physical activity? Which school characteristics are most effective to promote inclusion of children and young people with neurodisability in education and afterschool clubs? What is the long-term safety, effectiveness and sustainability of behavioural strategies and/or drugs (eg, melatonin) to manage sleep disturbance?
Attention Deficit Hyperactivity Disorder (Jacobson et al., 2016)	Is there a risk that medication with methylphenidate during childhood will lead to the development of drug dependence later in life? What are the effects of teacher support? What are the effects of multimodal therapy? Which of the two pharmaceuticals, atomoxetine or methylphenidate, is most effective, with fewer side effects? What are the effects of methylphenidate medication in substance abusers? What are the effects of parental support programmes? What are the effects of supported conversation? What are the effects of computer-aided working memory training? What are the effects of psychoeducative treatment? What are the effects of treatment of sleep disorders with melatonin?
Pediatric Obesity (McPherson et al., 2016)	Early and sustained engagement of families, rethinking determinants of obesity and health, maximizing impact of research, inclusive integrated interventions, evidence-informed measurement and outcomes, reducing weight biases
Systemic lupus erythematosus (SLE) (Tunnicliffe et al., 2017)	Improving service shortfalls, strengthening well-being, ensuring cost efficiency, minimizing family/community burden, severity of comorbidity or complications, reducing lifestyle disruption, fulfilling future goals
Pediatric Preventative Care (Lavigne et al., 2017)	Effective strategies for screening and prevention of mental health problems, interventions to increase physical activity in children, impact of daycare attendance on child health and development, effective interventions for obesity prevention in young children, interventions that promote social skill development, parental stress, screening methods for developmental delay in children, strategies for behavior management in children, nutritional factors that affect behavior in children, appropriate screen time (computer, iPad, tablets, phones, electronic gaming)
Sudden Infant Death Syndrome (SIDS) (Hauck et al., 2017)	Studying mechanisms leading to death and how they interact with environmental risk factors, Enabling best practice processes and systematic data collection for accurate classification of SUID deaths to inform research and prevention, Developing and evaluating new ways to make safe sleep campaigns more effective, Understanding to what extent social and cultural factors affect parental choice in sleep practices and responses to risk reduction campaigns, Identifying specific biomarkers to assist pathologists in determining the cause of death, Understanding the role of genetic factors in SUID risk, Understanding what mechanisms underlie SUID risk at different ages, Conducting additional research on the

	role of abnormal or immature brain anatomy and physiology, Better understanding of the practice of sharing any sleep surface with an infant, notably how it interacts with other, factors to make it more or less risky, Identifying what factors are associated with SUID in which all aspects of recommended risk reduction have been followed
Pediatric pain and Palliative Care (Baker et al., 2015; Liossi et al., 2017; Malcolm et al., 2008, 2009)	Develop and evaluate strategies to help families make difficult care decisions. Compare outcomes for patients and families who have early access to programs with outcomes for patients and families referred late in the illness trajectory, Develop strategies to teach HCPs how to help parents make difficult decisions and evaluate the impact of this education on relevant clinical outcomes, Develop and validate evidence-based practice guidelines in PPC, Study strategies for integrating quality palliative care practices into the ongoing care of seriously ill children in a variety of care settings, and evaluate the impact of these strategies on relevant care processes and outcomes, Study strategies designed to help parents understand what to expect and to prepare them for the possibility of death, Study the benefits (eg, costs, satisfaction with care, QOL, burden of care) of palliative care programs and services for patients, siblings, and parents in diverse clinical contexts, Study the role of the child in making treatment decisions about his or her palliative and EOL care, Test symptom interventions (eg, pain, dyspnea, fatigue, nausea, constipation, disturbed sleep, anxiety, depression) for infants, children, and adolescents, Compare outcomes for families who pursue aggressive treatment with curative intent in the care of children with advancing illness with outcomes for families who pursue supportive, non–cure–directed care, Study barriers to pediatric hospice and palliative care and strategies to overcome those barriers locally, nationally, and internationally (ie, access, referral, cultural, religious, communication, implementation), Study the impact of symptom-control interventions on the child’s level of comfort, function, and QOL, Develop strategies to teach pediatric hospice and palliative care and evaluate the impact of this education on relevant outcomes, Establish core quality indicators for pediatric hospice and palliative care and evaluate the effect of these measures on care processes and outcomes, Develop/evaluate strategies to help parents communicate with their child about the child’s life-threatening illness and the possibility of death, Develop and evaluate strategies to support family members of seriously ill children, Study the experience of children with incurable illness whose lives are prolonged with invasive artificial life sustaining therapies, Study how to resolve conflicts (ie, ethical dilemmas) that arise in the care of seriously ill children, Develop and implement valid symptom assessment tools in the care of infants, children, and adolescents, Study the effect of bereavement care interventions on relevant outcomes, Children with chronic pain with neuropathic characteristic, Pain in Pre-school children post-surgery, Children with acute pain at home, Children with chronic pain, Children receiving palliative care on long-acting opiate stable dose with poorly controlled pain, Pain and palliative care in pre-term and low birth weight infants, Awareness of children’s hospice care, Improving access to children’s hospice care, Supporting families who are new or potential users of hospice, Community care, Hospice and respite care needs of young people, Supporting the wider family, Needs of young people with life limiting conditions from their perspective, Identify services available to relieve pain and symptoms, Identify the symptoms experienced by children to develop management and control strategies, Explore ways to provide additional medical cover, Find out the specific care needs of young people

	(16 +) with limited cognitive abilities, Work together with local services to review and develop outreach respite teams across Scotland, Find out what families want from the hospice with regard to end-of-life and bereavement care, Identify ways to promote the awareness of the hospice, and the wide range of services it offers to children and families, Find out what the needs of siblings (brothers and sisters) are throughout each stage of a child's illness including bereavement support
Hematology, oncology, immunology and infectious diseases (Aldiss et al., 2018; Clinton-McHarg et al., 2010; Medlow and Patterson, 2015; Soanes et al., 2000)	What psychological support package improves psychological well-being, social functioning and mental health during and after treatment? What interventions, including self-care, can reduce or reverse adverse short- and long-term effects of cancer treatment? What are the best strategies to improve access to clinical trials? What General Practitioner or young person strategies, such as awareness campaigns and education, improve early diagnosis for young people with suspected cancer? What are the best ways of supporting a young person who has incurable cancer? What are the most effective strategies to ensure that young people who are treated outside of a young person's Principal Treatment Centre receive appropriate practical and emotional support? What interventions are most effective in supporting young people when returning to education or work? How can parents/carers/siblings/ partners be best supported following the death of a young person with cancer? What is the best method of follow-up and timing which causes the least psychological and physical harm, while ensuring relapse/complications are detected early? What targeted treatments are effective and have fewer short- and long-term side-effects? What support/information do shared-care hospitals want from the Regional Centre? Whether parents who have English as their second language really understand their child's illness and protocols? Quality of life from the children's perspective, Whether families are being given adequate information to give informed consent? What are the options for children regarding their future fertility? Are children/teenagers offered opportunities to bank sperm/ovaries and are these opportunities equal? If sibling donors are getting adequate support and preparation before, during and after bone marrow transplant, Whether effective and appropriate negotiation takes place between nursing staff and families Information giving to families at diagnosis--who tells the child about their cancer, If parent-held records assist in communication between the Regional Centre and District General Hospital, Identify risk and resilience, Develop psychosocial measures, Translate research into practice, Treatment centre/care delivery, Peer support/social activities, Physical/psychological therapy, Online interventions/technology Information/education, Finding a cure, Understanding the genetic basis of conditions, Understanding the relationship between a patients' genetics and their treatment, Development of predictive tests, Trials of new formulations of existing medicines, Trials of new medications, Trials of psychosocial therapies, Differences in researchers and patients overall priorities, Peer support, Support for parents and siblings, Transition from paediatric to adult care, Assessment of psychosocial aspects of their rheumatic condition, Reliance on specialist care, Interest in other models of care and services, Learning from good practice in terms of health services, Raising awareness of condition in schools and workplaces, Raising awareness of invisible conditions, Developing patient databases/biobanks, Cancer Control & Outcomes Research, Anti-cancer Treatment, Early Detection, Diagnosis & Prognosis, The need for holistic treatment plans, pain and symptom

	management, greater understanding of lifestyle factors such as physical rehabilitation, Survivorship, Testing the Benefits of Physical or Psychological Therapies, Youth Healthcare Needs & Professional Development, Prevention, Screening & Early Detection and Finished Treatment, How well nausea and vomiting are controlled for patients having chemotherapy, following discharge, assessing and treating children's nutritional needs, The most appropriate way to assess antibiotic levels, Ward-based isolation procedures using a multi-disciplinary approach involving infection control, nurses, play specialists, Methods available to pre-medicate children, Retaining staff what incentives do staff need e.g. Study time, time owing, The incidence of stress/burnout on the unit and support available, The most effective form of documenting.
Pediatric Emergency Medicine (Bialy et al., 2018)	Mental health, pain and sedation, practice tools (e.g., clinical practice guidelines, order sets, protocols, algorithms), quality of care delivery, Resource utilization, major/multisystem trauma, clinical prediction rules, emergency department communication, antibiotic stewardship, bronchiolitis/preschool wheeze, febrile young infant, patient safety, patient/family adherence with recommendations, traumatic brain injuries
Preterm Birth (Franck et al., 2018)	How does a mother's stress affect the baby? What are the most effective ways to improve patient-provider communication? What is the most effective care for pregnancy and high-risk pregnancy? What causes Sudden Infant Death Syndrome? Does the type of insurance you have determine the type of care that you get, or the quality of your care and is care different based on insurance status or race? What could make hospital visits and in-hospital stays easier for families and what supports are most helpful for moms with children at home? What medicines are safe to take during pregnancy? How do birth plans help and how can the health care team better follow a woman's birth plan? How do health care providers decide to involve Child Protective Services during pregnancy care when abuse and neglect are not clearly present? Could experienced moms be used more effectively for breastfeeding support?
Cystic Fibrosis (Rowbotham et al., 2018)	What are the effective ways of simplifying the treatment burden? How can we relieve gastro-intestinal (GI) problems? What is the best treatment for non-tuberculous mycobacterium (NTM)? Which therapies are effective in delaying or preventing progression of lung disease in early life? Is there a way of preventing Cystic Fibrosis related diabetes (CFRD)? What effective ways of motivation, support and technologies help people with Cystic Fibrosis improve and sustain adherence to treatment? Can exercise replace chest physiotherapy? Which antibiotic combinations and dosing plans should be used for Cystic Fibrosis exacerbations and should antibiotic combinations be rotated? Is there a way of reducing the negative effects of antibiotics eg, resistance risk and adverse symptoms? What is the best way of eradicating Pseudomonas aeruginosa?
Post-Pediatric Intensive Care Unit (Manning et al., 2018)	Family support, communication and information improvement between patients and families and HCPs, support during transitioning form hospital to home
Learning difficulties (Lim et al., 2019)	What is the best educational and community environment? How can multiple types of professionals work together with parents and carers to improve identification, diagnosis, interventions and treatments and achieve the best outcomes? Which early interventions are effective at what ages and stages are they best introduced and what are the long-term outcome? What knowledge, skills and training do health, social work and 'third sector' (e.g. charities and support

	services) professionals need to understand the best support to give? How can parents, carers, brothers and sisters and extended families be best supported to achieve their best quality of life before, during and after the diagnosis in home, school and community contexts? How can we best identify early features, symptoms and signs of learning difficulties? What is the best way to assess learning difficulties? Which strategies are effective in preventing stigma and bullying? Which strategies are effective in helping children live independent lives?
Pediatric Inflammatory Bowel Disease (IBD) (Grant et al., 2019)	Causes, preventions, the role of diets in the management of pediatric disease, trigger flare ups, improvement of non-invasive biomarkers of IBD endoscopic activity, increase awareness, long term effects of medications, early diagnosis and impact, access to psychological/mental health support, approach to diagnosis
Childhood Chronic Conditions (Lopez-Vargas et al., 2019)	What support networks are in schools? How can we better address the needs of children and families? What is the mental health outcome of a child with chronic disease, and does this reflect parental coping strategies? How can the system improve connection and support between parents/families of children with chronic disease? What is the best way to improve communication programs to empower families and children? What training/educational programs optimize functional outcomes in children? What are the barriers to health interventions and research? What are the triggers of anxiety? What does knowledge transfer, support and empowerment look like for families? What is the best model to coordinate care across health and education sectors? What do children and adolescents identify as important outcomes? What is the short-term/long-term impact of the education system and processes on children? What are the early-life determinants of long-term outcomes? What communication and care frameworks currently exist that would increase the quality and consistency of chronic care and outcomes? What type of education/training programs for families are the most effective to improve self-management and quality of life? What interventions help children with chronic conditions develop resilience? What interventions empower and support carers? How can we lessen the social impact of the stigma/ knowledge gaps? What are the most effective ways to engage children in their own management of their chronic illness? What are the different coping strategies used by parents and caregivers to overcome difficult situations? What programs are available in schools to improve social and educational outcomes for students who frequently miss school? What is the best way to enable teenagers to manage maintain and optimize adherence to treatment?
Pediatric Patient Safety (Hoffman et al., 2019)	Identify how to achieve high reliability, compare the effectiveness of strategies to create and maintain a culture that ensures patient safety, develop and test models to improve the speed and accuracy of predicting patient deterioration, develop processes and methods to encourage open communication between families and doctors, nurses, and other health care workers, compare the effectiveness of strategies to shorten the time and improve the accuracy of the detection of sepsis, identify the most common types of diagnostic errors and delays in pediatrics, and develop and test interventions to prevent diagnostic errors, evaluate improvement strategies in response to early warnings of patient deterioration, identify and compare best practices for safe communication between all health care workers caring for patients as they move between different health care settings, identify and compare best practices to ensure that the right patient is given the right treatment always and in all settings, compare the effectiveness of interventions to achieve accurate and complete

	medication lists in all patients in all settings, identify the unique safety needs of children with complex chronic conditions across health care settings and in transitions between settings; identify interventions for safe care and evaluate the impact, compare the impact of communication strategies on patient safety, identify interventions that decrease the rates of infection caused by health care, identify the best methods to measure situational awareness, identify best practices for ideal team work and evaluate their impact on patient safety, evaluate the impact of interventions on situational awareness and patient safety, identify and compare best practices to ensure hand-washing all of time in all health care settings and evaluate the impact on infection, Identify and assess interventions to improve the frequency and quality of staff safety reports, including unsafe conditions that do not actually cause harm, identify the types and rates of injuries caused by health care in the outpatient setting, develop and test strategies to reduce harm, develop and test programs to prevent mistakes in giving shots to all children in all settings, Evaluate the safety of care by allied health staff and develop and test strategies to improve safety, identify practices to reduce the rates of upper respiratory and gastrointestinal tract infection in the 2 wk after a visit to a hospital or clinic with a shared waiting room, Identify best practices to decrease the time spent in waiting rooms for patients who are immunocompromised, determine if less time in waiting rooms causes less infections in the 2 wk after a clinic visit
Cleft lip and palate (The James Lind Alliance, accessed 2020)	What types of psychological intervention (individual therapy, community or school based) and at what time (from diagnosis to adulthood) are most helpful for patients with a cleft of the lip and/or palate and their families? What are the educational, employment and personal (eg relationships) outcomes during childhood, adolescence and in the long term? What is the best protocol for primary repair of both the lip and palate, including technique/timing and sequence? In individuals with a cleft of the lip and/or palate when is the most effective age to begin speech therapy? What is the best treatment for otitis media with effusion (glue ear)? Can stem cells be used to improve palate repair (both primary and secondary)? What interventions would enhance the educational outcomes for children? What is the impact of having a baby born with a cleft of the lip and/or palate on maternal/child attachment? What is the best way to manage infants with a cleft of the lip and/or palate undergoing primary surgery before, during and after hospital Eg. Fluids, pain control, antibiotics, probiotics, arm splints, feeding practices? What are the genetic and environmental causes of clefts of the lip and/or palate? How can we improve the diagnosis of cleft palate (without cleft lip)? What is the best way to prevent tooth decay in children with a cleft of the lip and/or palate?
Paediatric Lower Limb Surgery (The James Lind Alliance, accessed 2020)	What are the best ways to measure outcome following lower limb orthopaedic surgery in children? What should children's rehabilitation following orthopaedic surgery to the lower limbs include, how long is it expected to last and how does it affect the result of treatment? What is the short-term and long-term clinical and cost effectiveness of orthopaedic lower limb surgery for children with Cerebral Palsy who can walk? What are the short term and long-term outcomes of surgery compared to non- surgical care in the treatment of Perthes disease? What is the role of pre-operative rehabilitation in children presenting with lower limb orthopaedic conditions? What is the short-term and long-term clinical and cost effectiveness of Selective Dorsal Rhizotomy (SDR) in children with Cerebral Palsy who can walk? Can

	surveillance and non-surgical treatment (physiotherapy, botulinum toxin injections, functional electrical stimulation, orthotics, casting) prevent the development of deformity requiring surgery in children with Cerebral Palsy? What is the best method of screening for Developmental Dysplasia of the Hip (DDH) in terms of clinical and cost effectiveness? What are the best strategies to optimise communication of information between patients/carers and HCPs in order to enable shared decision making? What is the best management for hip displacement in children with Cerebral Palsy?
Children and Youth with Special Health Care Needs (CYSHCN) (Coller et al., 2020)	Home environment as a health determinant; interventions to support caregiving at home; best practices for caregiver education and peer-to-peer support, Technologies to complement caregiving and communication; dissemination or implementation of technology, Workforce capacity and expertise; CYSHCN, health care, and community links; durable equipment, education, and dental care, Payment model influence on quality, access, and outcomes; compensation for families, providers, and community services, Key ingredients of effective models; family partnership, team efficiency, provider retention, and culturally sensitive care, Shared decision-making and goal setting; generalizable CYSHCN health screening and co-management approaches, Interventions to reduce consequences of social determinants, rural environments, mental health, and extreme complexity; health outcomes meaningful to families, Interventions to promote family physical and mental health, resilience, and self-care; health system influence on family caregiver health, Rare disease cohorts; CYSHCN as research team members; marketing findings to research consumers
